# Patients With Short PFS to EGFR-TKIs Predicted Better Response to Subsequent Anti-PD-1/PD-L1 Based Immunotherapy in EGFR Common Mutation NSCLC

**DOI:** 10.3389/fonc.2021.639947

**Published:** 2021-03-11

**Authors:** Sangtian Liu, Fengying Wu, Xuefei Li, Chao Zhao, Yijun Jia, Keyi Jia, Ruoshuang Han, Meng Qiao, Wei Li, Jia Yu, Fei Zhou, Anwen Xiong, Bin Chen, Jue Fan, Shengxiang Ren, Caicun Zhou

**Affiliations:** ^1^ Department of Medical Oncology, Shanghai Pulmonary Hospital, Tongji University Medical School Cancer Institute, Tongji University School of Medicine, Shanghai, China; ^2^ Department of Lung Cancer and Immunology, Shanghai Pulmonary Hospital, Tongji University Medical School Cancer Institute, Tongji University School of Medicine, Shanghai, China; ^3^ Department of Bioinformatics and Data Science, Singleron Biotechnologies, Nanjing, China

**Keywords:** targeted therapy, immunotherapy, programmed cell death ligand-1, EGFR-tyrosine kinase inhibitors (EGFR-TKI), epidermal growth factor receptor

## Abstract

**Background:**

Despite disappointing outcomes from immuno-monotherapy, studies reported that NSCLC patients with EGFR mutation may possibly benefit from combined immunotherapy. Whether the response to prior EGFR-TKI has association with the outcomes of subsequent immunotherapy remains unclear.

**Patients and Methods:**

Advanced NSCLC patients with resistance to EGFR-TKIs and received ICI treatment from January 2016 to June 2019 were retrospectively analyzed. Single cell sequencing and flow cytometry were conducted to explore the difference of cell components in tumor microenvironments (TME). A 1:3 matched case–control study was conducted to compare the clinical effects of combined immunotherapy with standard chemotherapy as second-line treatment.

**Results:**

Fifty-eight patients treated with anti-PD-1/PD-L1 based immunotherapy behind EGFR-TKI treatment were enrolled. Correlation analysis showed TKI-PFS had a significantly negative association with corresponding IO-PFS (r = −0.35, p = 0.006). TKI-PFS cutoff 10 months had the most significant predictive function for posterior immunotherapy and was validated to be an independent predictor by uni- and multivariate analyses. Kaplan–Meier analysis showed that patients with TKI-PFS less than 10 months had significantly prolonged IO-PFS and higher ORR than those with long (median PFS, 15.1 *vs* 3.8 months; HR, 0.26, p = 0.0002; ORR, 31.8 *versus* 10%, p = 0.04). Single cell RNA-seq revealed that the cell components were varied among patients after treatment with EGFR-TKI. Patients with short TKI-PFS demonstrated a relatively higher proportion of CD8 effector cells and lower ratio of M2 like macrophage to M1 like macrophages, which was validated by flow cytometry. Case–control study demonstrated that combined immunotherapy achieved significantly longer PFS (HR, 0.51, 95% CI: 0.31–0.85, p = 0.02), longer OS (HR, 0.48, 95% CI: 0.26–0.89, p = 0.05) and higher ORR (33.3 *vs* 10.0%, p = 0.02) than traditional chemotherapy for patients with short TKI-PFS.

**Conclusion:**

Patients with short TKI-PFS conferred better response to immunotherapy than those with long. The status of TME were different among those two populations. Combined ICI treatment could promisingly be a better choice than classical chemotherapy in second-line setting for patients with short TKI-PFS and no T790M mutation. Underlying mechanisms need to be further explored.

## Introduction 

Lung cancer remains to be the most frequent and deadly malignant disease, with 1.6 million tumor-related deaths annually worldwide. Non-small-cell lung cancer (NSCLC) represents approximately 85% of all new lung cancer diagnoses (1). For inoperable advanced NSCLC patients with the epidermal growth factor receptor (EGFR) sensitive mutation, EGFR-tyrosine kinase inhibitors (EGFR-TKIs) have been recommended as the first line choice (2–5). However, drug acquired resistance is inevitable. The most acceptable subsequent treatment for those without EGFR T790M mutation was platinum-based cytotoxic chemotherapy; nevertheless, the clinical benefit was limited (6).

Immune checkpoint inhibitors (ICIs), especially antibodies targeting programmed death-1 (PD-1) and programmed death ligand-1 (PD-L1), provided a new paradigm against cancer and were considered as promising anti-tumor treatments in NSCLC (7–10). Despite its marked successful applications in clinical practice, the efficacy and responsiveness of ICI monotherapy in patients with EGFR sensitive mutation were disappointing (7, 10–13). However, there was still a glimmer of hope when combining the conventional chemotherapy or antiangiogenic therapy with ICI treatment demonstrated promising results among those population with positive driver genes (14, 15). The PROLUNG comparing pembrolizumab plus docetaxel *versus* docetaxel alone in pretreated NSCLC patients involved 25 patients with EGFR/anaplastic lymphoma kinase (ALK) alteration and found that the combination immunotherapy improved the objective response rate (ORR) and progress-free survival (PFS) in patients with EGFR variation (14). The subgroup analysis of Impower150 showed that patients with EGFR positive mutations could benefit from the atezolizumab combined therapy (10). Same as the former, our previous study achieved consistent outcomes when comparing toripalimab (anti-PD-1 mono-antibody) combination therapy with single chemotherapy in advanced NSCLC patients progressed on EGFR-TKIs (15). In addition, previous studies found that high tumor mutation burden (TMB) and PD-L1 expression, which were considered to predict better outcomes from immunotherapy, were more likely presented in patients with relatively shorter PFS to EGFR-TKI (16, 17). However, no research has investigated the predictive function of PFS to EGFR-TKI for posterior immunotherapy.

Hence, in this study, we aim to analyze the association of targeted therapy with posterior ICI treatment to explore which subgroup of EGFR mutated patients could most likely benefit from anti-PD-1/PD-L1 based immunotherapy after progression on EGFR-TKI treatment.

## Patients and Methods

### Study Population

EGFR mutated NSCLC patients treated in Shanghai Pulmonary Hospital from January 2016 to June 2019 were retrospectively screened. All enrolled patients met the following inclusion criteria: they were aged ≥18 years; had histologically or pathologically confirmed stage IIIB or stage IV or recurrent NSCLC; confirmed EGFR-activating mutation (including 19DEL and L858R), treated with first- or second-generation EGFR-TKI, received anti-PD1/PD-L1 based mono- or combined immunotherapy in posterior lines, and had available medical records. Patients met following conditions were excluded: received immunotherapy before EGFR-TKIs target therapy, had EGFR T790M mutation at base line, treated with less than two circles of immunotherapies. The electric medical records were retrospectively reviewed; detailed clinicopathologic characteristics and clinical responses were collected.

### Molecular Analysis

Gene mutation analyses were performed at the Thoracic Cancer Institute, Tongji University Medical School, in Shanghai. The detailed process was the same as described in our previous studies (18, 19). Briefly, DNA was extracted from the tissue by using the DNeasy Blood and Tissue Kit or the QIAamp DNA FFPE Tissue Kit (both from Qiagen, Hilden, Germany). The common EGFR mutations, including exon 19 deletion (19DEL), exon 21-point mutation (L858R), and exon 20-point mutation (T790M), were tested by using amplification refractory mutation system (ARMS+) (Human EGFR Gene Mutation Quantitative Detection Kit, Genosaber Biotech, Shanghai, People’s Republic of China). After EGFR-TKI resistance, re-biopsy was recommended for the detection of T790M. Only those with poor status, risky lesions, or the patient and its family refused re-biopsy, would take the evaluation by liquid biopsy. PD-L1 expression in tumor specimens obtained after the acquisition of EGFR-TKI resistance was determined by immunohistochemistry (kit with clone 22C3 and clone 28-8, Agilent Technologies). PD-L1 positivity was defined as tumor proportion score (TPS) cutoff of 1%.

### Single Cell RNA Sequencing

#### Sample Collection and Preparation

Eighteen re-biopsy samples were collected from advanced NSCLC patients after they progressed on first- or second-generation EGFR-TKIs from 2019 to 2020 in Shanghai Pulmonary hospital. The fresh tumor tissue was stored in the GEXSCOPE™ tissue preservation solution (Sigleron) and transported to the Singleron lab as soon as possible on ice. The tissue was washed with Hanks Balanced Salt Solution (HBSS) (Gibco™, 14025092) for three times, then minced into 1–2 mm pieces and digested with 2 ml GEXSCOPE™ Tissue Dissociation Solution (Singleron) at 37°C for 15 min. After that, a 40-µm sterile strainer was used to filter the sample, and the single cell suspension was centrifuged at 1,000 rpm for 5 min. After discarding the supernatant, the sediment was resuspended with 1 ml PBS (Hyclone, SH30256.01). When there was a need to remove red blood cells, 2 ml GEXSCOPE™ red blood cell lysis buffer (Singleron) was added at room temperature for 10 min. Then, the solution was centrifuged at 500 × g for 5 min, and the sediment was resuspended in PBS. The sample viability was microscopically evaluated by trypan blue staining (Sigma, T6146).

#### Single Cell RNA Sequencing, Quantification and Statistical Analysis

Single cell suspension was prepared with PBS (Hyclone, SH30256.01) in concentration of 1 × 10^5^ cells/ml. Then, the suspension was loaded onto microfluidic devices, and scRNA-seq libraries were established according to Singleron GEXSCOPE™ protocol by GEXSCOPE™ Single-Cell RNA Library Kit (Singleron Biotechnologies) (20). Individual libraries were diluted to 4 nM and pooled for sequencing. Pools were sequenced on Illumina HiSeq X with 150 bp paired end reads. An internal pipeline was used to process raw reads to generate gene expression profiles. Briefly, cell barcode and UMI were extracted after filtering read one without poly T tails. Adapters and poly A tails were trimmed (fastp V1) before aligning read two to GRCh38 with ensemble version 92 gene annotation (fastp 2.5.3a and featureCounts 1.6.2) (21). Reads with the same cell barcode, UMI, and gene were grouped together to calculate the number of UMIs per gene per cell. The UMI count tables of each cellular barcode were used for further analysis. Seurat program was used for analyzing RNA-Sequencing data, including cell type identification and clustering analysis (22, 23) (http://satijalab.org/seurat/, R package, v.3.0.1). UMI count tables were loaded into R using read.table function. The parameter resolution was set to 0.8 for FindClusters function to clustering analysis.

### Flow Cytometry

Tumor specimens were harvested at the time of resistance to first- or second-generation EGFR-TKI by re-biopsy. Single-cell suspensions were yielded by grinding with a syringe piston through a 40 um filter. Erythrocytes were lysed in Red Blood Cell Lysis Buffer (Sigma, R7757). Approximately 1 × 10^5^ cells were used for flow cytometry staining. For INF-*γ* detection, the cells were stimulated with Leuko Act Cktl With GolgiPlug (BD Pharmingen, 550583) for 4–6 h in advance. Then, cells were blocked with Fc block (BD Pharmingen: #564219) for 15 min at 4°C. After that, surface staining was performed in 1× PBS with 2% fetal bovine serum (FBS) for 30 min at 4°C, for lymphocyte panel: CD3 (BD Pharmingen, 560835), CD8 (BD Pharmingen, 565166), CD4 (BD Pharmingen, 563877), and for myeloid panel: CD45 (BD Pharmingen, 563204), CD68 (BD Pharmingen, 562117), CD11b (BD Pharmingen, 564985), CD33 (BD Pharmingen, 745556), HLA-DR (BD Pharmingen, 562331) and CD86 (BD Pharmingen, 557344). Subsequently, cells were fixed and permeabilized with Perm/Fix solution (Invitrogen, 00-5521-00) for 45 min in the dark at 4°C. Then, lymphocyte panel was intracellularly stained with INF-*γ* (BD Pharmingen, 563416) and Foxp3 (BD Pharmingen, 560046), and myeloid panel stained with CD206 (BD Pharmingen, 561763) anti-human antibodies. Data acquisition was conducted using a Beckman CytoFLEX S and analyzed using FlowJo software version 10.0 (Tree Star, Inc.). Dead cells were excluded from analysis by using fixable viability stain 780 (fvs780, BD Pharmingen,565388) as per manufacturer’s instructions.

### Statistical Analysis

The categorical variables were compared by Chi-square test or Fisher exact test when needed. Pearson correlation coefficients were computed for correlation analysis. Kaplan–Meier curve and two-sided log-rank test were used for univariate survival analysis. For uni- and multivariate survival analyses, Cox proportional hazards model was used to calculate the hazard ratios (HRs) and corresponding 95% confidence intervals (CIs). Disease evaluation was defined by the Response Evaluation Criteria in Solid Tumors guidelines 1.1 (RECIST1.1). PFS was calculated from the date of the corresponding treatment initiation to the date of systemic progression or death and was censored at the date of the last tumor assessment (when carried out). Overall survival (OS) was calculated from the time of randomization to death caused by any cause. ORR was equal to the sum of complete response (CR) plus partial response (PR). The Mann–Whitney U test was used to find markers that are significantly different (p < 0.05) between the patients with short or long TKI-PFS group. All statistical analyses were performed using the SPSS statistical software, version 22.0 (SPSS Inc., Chicago. IL). The figures were drawn by GraphPad prism 7.03 and Adobe photoshop CS4. *p* values were two-sided and considered significant if less than 0.05.

## Results

### Patients’ Demographics, Clinical Characteristics, and the Correlation of Prior Targeted Therapy With Posterior Immunotherapy

**Figure 1:**
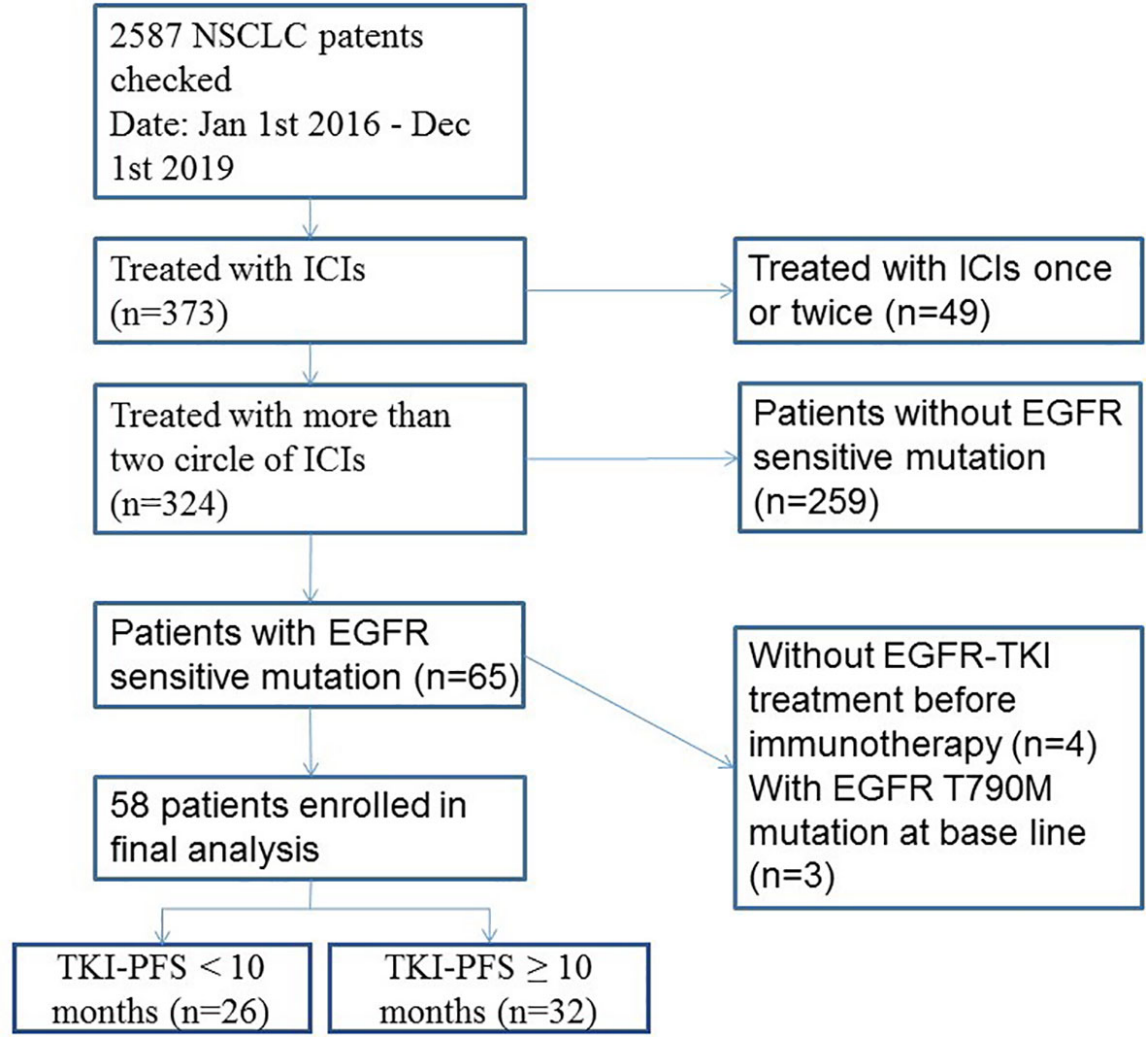
Flowchart of cohort with EGFR mutation and immunotherapy. ICIs, immune checkpoint inhibitors.

In this study, 58 advanced NSCLC patients with EGFR sensitive mutation and treated with ICI based immunotherapy were enrolled (**Figure 1**). The median age was 58.8 years old. Males accounted for 56.9% (33/58), and 84.5% (49/58) were never smoker. Among them, 43.1% (25/58) of patients received ICIs in second-line treatment. Most of them (54/58, 93.1%) had combined chemotherapy (42 of them with pemetrexed, four of them with docetaxel and eight of them with albumin-bound paclitaxel) and four of them combined with antiangiogenic therapy; no patients combined with chemotherapy and antiangiogenic therapy at the same time (**Table 1**).

**Table 1:** Clinical characteristics.

Characteristics	All cases	EGFR-TKIs’ PFS<10m	EGFR-TKIs’ PFS≥10m	p
Total, n	58	26	32	
Median age(range), y	58.8 (21–80)	57.9 (21–76)	59.6 (25–80)	
Age group, n (%)				0.128
<65 years	39 (67.2)	20 (76.9)	19 (59.4)	
≥65 years	19 (32.8)	6 (23.1)	13 (40.6)	
Gender, n (%)				0.438
Male	25 (43.1)	12 (46.2)	13 (40.6)	
Female	33 (56.9)	14 (53.8)	19 (59.4)	
Smoking history, n (%)				0.365
Never-smoker	49 (84.5)	21 (80.8)	28 (87.5)	
Former/current smoker	9 (15.5)	5 (19.2)	4 (12.5)	
ECOG performance status, n (%)				0.119
0-1	53 (91.4)	22 (84.6)	31 (96.9)	
2	5 (8.6)	4 (15.4)	1 (3.1)	
Pathological classification, n (%)				0.611
Adenocarcinoma	54 (93.1)	24 (92.3)	30 (93.8)	
NSCLC NOS	4 (6.9)	2 (7.7)	2 (6.2)	
TNM stage, n (%)				0.389
III	4 (6.9)	1 (3.8)	3 (9.4)	
IV	54 (93.1)	25 (96.2)	29 (90.6)	
EGFR mutation type, n (%)				0.511
19DEL	28 (48.3)	13 (50.0)	15 (46.9)	
L858R	30 (51.7)	13 (50.0)	17 (53.1)	
Required T790M mutation, n (%)				0.356
Yes	13 (22.4)	4 (15.4)	9 (28.1)	
No Unknown	37 (63.8) 8 (13.8)	17 (65.4) 5 (19.2)	20 (62.5) 3 (9.4)	
Type of EGFR-TKIs, n (%)				0.512
Gefitinib	30 (51.7)	11 (42.3)	19 (59.4)	
Erlotinib	15 (25.9)	7 (26.9)	8 (25.0)	
Icotinib	13 (22.4)	8 (30.7)	5 (15.7)	
Best response to EGFR-TKI, n (%)				0.005
PR	40 (68.9)	13 (50.0)	27 (84.4)	
SD/PD	18 (31.1)	13 (50.0)	5 (15.6)	
Distant metastasis, n (%)				0.389
Yes	54 (93.1)	25 (96.2)	29 (90.6)	
No	4 (6.9)	1 (3.8)	3 (9.4)	
No. of immunotherapy line, n (%)				0.245
2	25 (43.1)	13 (50.0)	12 (37.5)	
≥3	33 (56.9)	13 (50.0)	20 (62.5)	
Treatment regimen, n (%)				0.587
*α*PD-1/PD-L1 monotherapy	7 (12.1)	4 (15.4)	3 (9.4)	
*α*PD-1/PD-L1 + Chemo	47 (81.0)	21 (80.8)	26 (81.3)	
*α*PD-1/PD-L1 + Apatinib	4 (6.9)	1 (3.8)	3 (9.4)	
PD-L1 expression, n (%)				0.186
Not detected	24 (41.4)	12 (46.2)	12 (37.5)	
Negative	17 (29.3)	5 (19.2)	12 (37.5)	
1–49%	11 (18.9)	6 (23.1)	5 (15.6)	
≥50%	6 (10.4)	3 (11.5)	3 (9.4)	
Local therapy, n (%)				0.611
Yes	11 (19.0)	5 (19.2)	6 (18.8)	
No	47 (81.0)	21 (80.8)	26 (81.3)	

ECOG, eastern cooperative oncology group; EGFR-TKIs, epidermal growth factor receptor; NSCLC, non-small-cell lung cancer; NOS, not otherwise specified; No, number; Chemo, chemotherapy.

**Figure 2:**
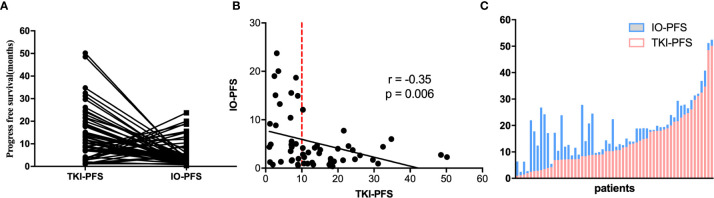
The correlation of TKIs-PFS with IO-PFS. **(A)** The one-to-one matched TKI-PFS in former line and IO-PFS in posterior line; **(B)** The distribution of TKIs-PFS and IO-PFS in cartesian coordinate system; **(C)** the waterfall plot of TKI-PFS with corresponding IO-PFS.

The median PFS of EGFR-TKIs targeted therapy (TKI-PFS) and posterior anti-PD-1/PD-L1 based immunotherapy (IO-PFS) was 10.4 and 5.5 months respectively (**Supplementary Figure 1**). When connecting TKI-PFS with corresponding IO-PFS, an inverse relationship was observed (**Figure 2A**). Correlation analysis showed that the TKI-PFS had a significantly negative association with the corresponding IO-PFS, with Pearson r = −0.35 (p = 0.006) (**Figure 2B**). The waterfall plot displayed that long IO-PFS was mainly distributed in the part of patients with short TKI-PFS and dramatically decreased in the part of patients with longer TKI-PFS (**Figure 2C**).

### The TKI-PFS Cutoff 10 Months Predicted PFS and ORR for Posterior ICI Treatment

**Figure 3:**
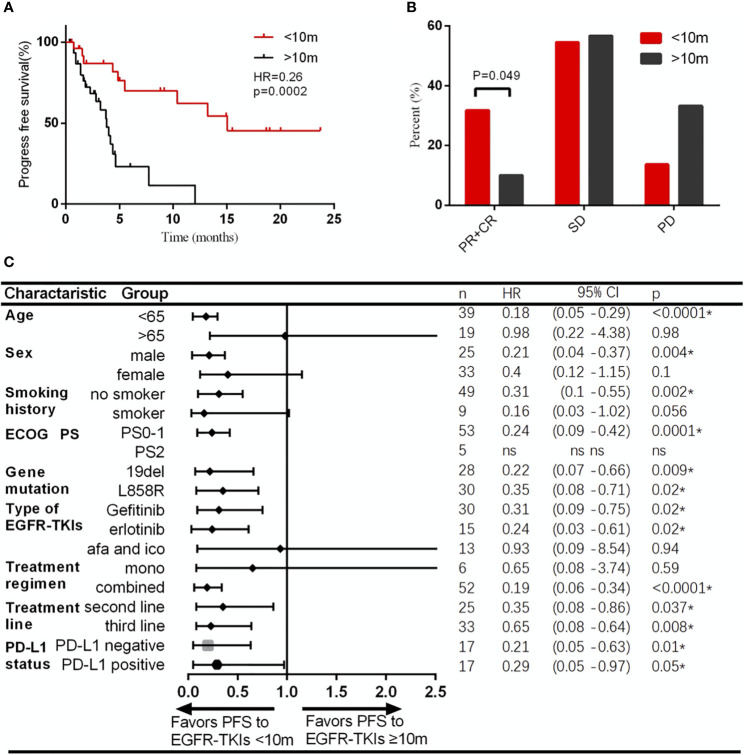
Patients with TKI-PFS less than 10 months had better response to immunotherapy than those with longer TKI-PFS. **(A)** Patients with TKI-PFS less than 10 months had significantly prolonged PFS than those with longer TKI-PFS; **(B)** Patients with TKI-PFS less than 10 months had significantly higher ORR to immunotherapy than those with longer TKI-PFS; **(C)** Forest plot of subgroup analysis by baseline characteristics for IO-PFS in patients with short or long TKI-PFS. *p < 0.05.

To find out the most significant cutoff value of TKI-PFS to predict the clinical response of posterior immunotherapy, we tested every TKI-PFS cutoff at one-month interval and it turned out that when TKI-PFS cutoff is 10 months, it achieved the most statistical significance in predicting IO-PFS, with median IO-PFS of 15.1 *versus* 3.8 months respectively (HR, 0.26, 95% CI, 0.12–0.50, p = 0.0002) (**Figure 3A**). Hence, we divided all patients into short and long TKI-PFS groups at cutoff of 10 months in the follow-up study.

Uni- and multivariate analyses validated that short TKI-PFS was independently associated with better clinical outcomes from subsequent immunotherapy (**Table 2**). Meanwhile, the ORR of immunotherapy was significantly higher in the short TKI-PFS group than in the long TKI-PFS group with 31.8 *versus* 10% (p = 0.04) (**Figure 3B**).

**Table 2:** Uni- and multivariate analyses of clinical parameters on progress-free survival to immunotherapy.

Factors	Univariate analysis		Multivariate analysis	
	HR (95% CI)	p Value	HR (95% CI)	p Value
Sex (Female/Male)	0.89 (0.42–1.91)	0.77		
Age (<65/≥65)	0.82 (0.34–1.96)	0.66		
Smoking (Never/Smoking)	0.65 (0.25–1.74)	0.39		
PS (0–1/2)	0.49 (0.23–1.07)	0.07★	0.62 (0.26–1.44)	0.27
Histology (Adno/Non-adeno	0.57 (0.076–4.21)	0.58		
EGFR type (19DEL/L858R)	1.25 (0.59–2.65)	0.55		
EGFR T790M (positive/negative)	0.709 (0.273–1.841)	0.46		
Stage (III/IV)	0.67 (0.15–2.92)	0.59		
Distant metastasis (Yes/No)	0.67 (0.15–2.92)	0.59		
Regimen of EGFR-TKIs				
Gefitinib	1			
Erlotinib	0.71 (0.29–1.73)	0.45		
Icotinib	1.08 (0.31–3.82)	0.9		
PFS to EGFR-TKIs				
<10 months	1.00		1	
≥10 months	5.17 (2.05–13.0)	0.000***★	5.93 (2.21–15.89)	0.000***
No. of immunotherapy line (2/≥3)	1.36 (0.88–2.08)	0.16★	1.46 (0.94–2.27)	0.09
Regimen of immunotherapy, n(%)				
*α*PD-1 monotherapy	1		1	
*α*PD-1 + Chemo	0.48 (0.18–1.29)	0.15★	0.33 (0.11–0.96)	0.043*
*α*PD-1 + Apatinib	0.27 (0.03–2.35)	0.24	0.14 (0.015–1.24)	0.08
PD-L1 expression (Positive/Negative)	0.81 (0.31–2.11)	0.67		
Radiotherapy (Yes/No)	1.11 (0.46–2.66)	0.82		

Same as in **Table 1**. ★ < 0.02, ***p < 0.001.

Next, we conducted a subgroup analysis to take clinical characteristics into consideration. Generally, majority of subgroups favored patients with short TKI-PFS, especially those with age less than 65 years old, male, no smoker, PS0-1, 19DEL or L858R, with gefitinib or erlotinib, ICI combined with chemotherapy, and ICI treatment in second line or third and later achieved a significant superiority from immunotherapy (**Figure 3C**).

### The PD-L1 Expression and Required T790M Mutation

The degree of PD-L1 expression on tumor cells was shown to correlate with the treatment of anti-PD-1/PD-L1 antibodies (24). Previous studies reported that negative PD-L1 expression predicted worse clinical benefits from immunotherapy for EGFR mutated NSCLC patients (17, 25). To investigate the impact of PD-L1 on the response of subsequent ICI treatment, we retrospectively collected the information of PD-L1 expression level after disease progression on EGFR-TKIs. There were 34 patients detected for PD-L1 expression. Among them, 17 patients were positive with tumor expression level cutoff 1% (**Table 1**). The positive rate was 64.3% (9/14) in the short TKI-PFS group and 40% (8/20) in the long TKI-PFS group. There was no difference observed from ICI treatments between PD-L1 positive and negative population no matter in the whole cohort or cohort stratified by TKI-PFS [**Supplementary Figure 2A** **(i–iii)**]. Intriguingly, both in PD-L1 positive and negative cohorts, patients with short TKI-PFS had a statistically prolonged IO-PFS than those with long TKI-PFS ([**Supplementary Figure 2A** **(iv–v)**].

T790M mutation was considered to be related with immunotherapy, and those with positive T790M mutation were less likely to benefit from it when compared with T790M-negative patients (25). In our study, 50 patients were detected for resistance mechanisms after targeted therapy and eight patients had no data about it. Re-biopsy was conducted in majority of them with only seven patients taking the liquid biopsy. There were 13 patients with positive T790M mutation and 37 with negative status. Subgroup analysis was performed according to the status of T790M. It was highly similar to PD-L1 expression as no difference was observed between T790M positive and negative population whether in the whole cohort, cohort with short TKI-PFS, or with long [**Supplementary Figure 2B** **(i–iii)**]. However, patients with short TKI-PFS demonstrated superiority regardless of T790M status [**Supplementary Figure 2B** **(iv–v)**].

### Tumor Immune Microenvironments Explored by Single Cell RNA-Sequencing and Flow Cytometry

**Figure 4:**
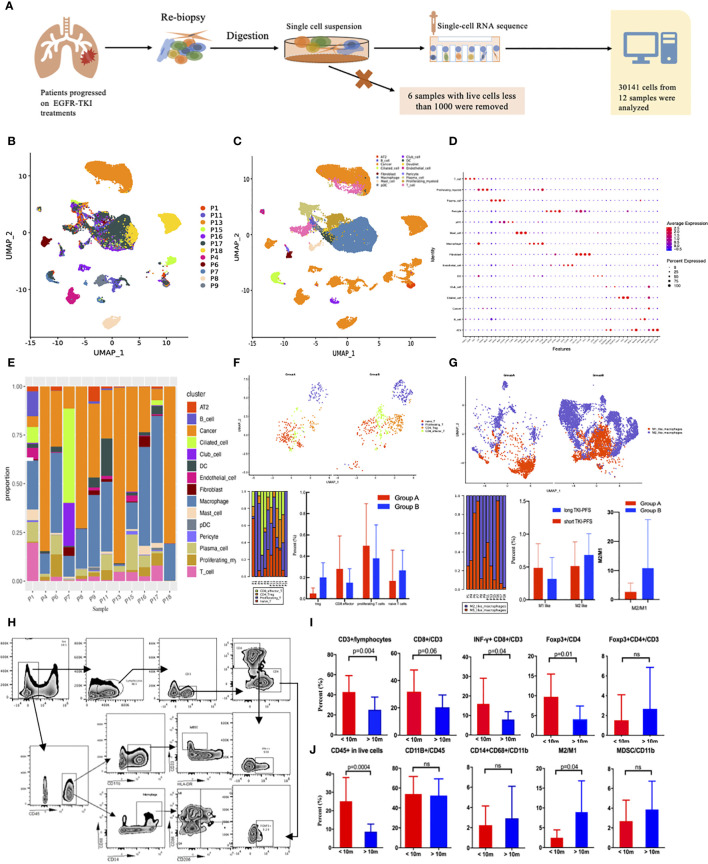
The analysis of single cell RNA sequencing and flow cytometry. **(A)** Flow diagram of single cell RNA sequencing; **(B)** Distribution of 12 samples by UMAP; **(C)** Distribution of 15 subtypes cells by UMAP; **(D)** Representative gene expression in 15 subclasses of cells; **(E)** Distribution of the 15 subclasses among 12 samples; **(F)** Subset analysis of T cells; **(G)** Subset analysis of macrophages; **(H)** the gating strategy for flow cytometry; **(I, J)**, Box plot of flow cytometry grouped by TKI-PFS for lymphocytes and myeloid cells (TKI-PFS <10 m = 13; TKI-PFS >10 m = 13). ns, none sense.

Since PD-L1 expression and T790M status could not completely explain the superiority of immunotherapy among patients with short TKI-PFS, other mechanisms must exist. Here, we successfully conducted single cell RNA-sequencing analysis to tumor specimens from NSCLC patients with pathologically confirmed lung adenocarcinoma (LUAD) or NSCLC and progressed on EGFR-TKIs treatment, which was illustrated in **Figure 4A**. After quality control and filtering steps, a total of 30,141 cells from 12 patients were eventually analyzed (**Figure 4B**). Fifteen major cell types were detected by leveraging canonical cell markers, including fibroblasts, endothelial cells, tumor cells, macrophages, T cells, B cells, mast cells, neutrophils, dendritic cells, and ciliated cells (**Figures 4C, D**). According to the findings above, we divided all patients into two groups by TKI-PFS cutoff of 10 months for further analysis. Among them, five (P1, P4, P6, P7, P8) had a TKI-PFS less than 10 months, which was defined as group A; and the other seven (P9, P11, P13, P15, P16, P17, P18) had a longer TKI-PFS (detailed information could be found in **Supplementary Table 1**), which was defined as group B. As we could see in **Figure 4E**, the major cell types were tumor cells and macrophages in all patients, and the proportion of immune related cells varied greatly among individuals (**Figure 4E**). To further analyze the immune functions of T cells and macrophages, more subsets were identified according to different roles in anti-tumor immune process. For T cells, there were four subtypes identified according to canonical cell markers, including CD8 effector T cells (CD3D, CD8A/B, GZMA, GNLY, NKG7), CD4+ Foxp3+ Tregs (CD3D, CD4, FOXP3, IL2RA), proliferating T cells (CD3D, MKI67, TOP2A, STMN1, CCNB2), and naïve T cells (CD3D, CCR7, SELL, LEF1, TCF7, IL7R) (**Figure 4F**, the top). And for macrophages, two subtypes were identified including M1 like macrophages (LYZ, CD68, IL1B, IL6, TNF) and M2 like macrophages (LYZ, MRC1, CD163, TGFB1, IL10, FN1) (**Figure 4G**, the top). When taking the subtypes of T cells and macrophages into consideration, it was found that patients in group A demonstrated relatively higher proportion of CD8+ effector T cells and proliferating T cells and had significantly lower proportion of Tregs and lower rate of M2 like macrophages to M1 like macrophages (**Figures 4F, G**, the below). To validate those findings, flow cytometry was performed among additional 26 re-biopsy specimens to analyze the major cell types of lymphocytes and macrophages, including 13 with TKI-PFS less than 10 months and 13 with longer (detailed information could be found in **Supplementary Table 2**). Gating strategies were displayed in **Figure 4H**. It turned out that patients with TKI-PFS less than 10 months had significantly higher proportion of CD45+ in live cells, CD3+ in lymphocytes, CD8+ in CD3+ T cells and INF-*γ*+CD8+ in CD3+ T cells infiltrated in the tumor microenvironment (TME) (**Figure 4I**). For the Treg detection, we defined foxp3+ cells in CD4+ T cells as the gating strategy, and it was shown that among CD4+ T cells, the proportion of foxp3+ cells was significantly higher in cohort with short TKI-PFS than in those with long TKI-PFS. However, when comparing Tregs in the whole CD3+ lymphocyte, there had no obvious difference between the two cohorts (**Figure 4I**). As for the macrophages, a significantly lower rate of M2 like to M1 like macrophages was observed in patients with short TKI-PFS; besides that, no difference was observed in CD11b+ myeloid cells and myeloid derived suppressor cells (CD33+HLA-DR- in CD11b+) (MDSCs) (**Figure 4J**).

### Comparison of Combined Immunotherapy With Conventional Chemotherapy as Second-Line Treatment in Patients Progressed on EGFR-TKIs

Up to now, the standard treatment for patients without EGFR T790M mutation after progression on prior line EGFR-TKI targeted therapy was cytotoxic chemotherapy (26). However, the response rate and clinical benefit were limited. Since we had observed benefits from combined immunotherapy in patients with short TKI-PFS, and the infiltration of immune cells in the TME also supports it theoretically, it was intriguing and worth exploring whether ICI combination treatment would be a better choice than traditional chemotherapy for those with short TKI-PFS and no T790M mutation. To validate it and rule out the influence of multi-line treatment before immunotherapy, we pull out the twenty-five patients who received IO combined treatment (Immune-cohort) in the second line after progression on EGFR-TKI as the match source to conduct a case–control study. Seventy-five patients who received platinum-based chemotherapy (Chemo-cohort) as second-line treatment were matched at a rate of 1:3 according to age, sex, EGFR mutated type and the initial time of diagnosis. The clinical characteristics were well balanced between the two cohorts as shown in **Table 3**. In the Immune-cohort, 24 patients received ICI combined with pemetrexed plus carboplatin and one patient received ICI combined with pemetrexed alone. As for the Chemo-cohort, 61 patients were treated with conventional chemotherapy (including 49 patients with pemetrexed plus carboplatin, five patients with pemetrexed plus cisplatin, four patients with mono-pemetrexed, one patient with gemcitabine plus carboplatin and two patients with pemetrexed plus oxaliplatin) and 14 patients received chemotherapy combined with angiogenesis therapy.

**Table 3:** Clinical characteristics of patients in case–control study.

Characteristics	All cases	Immunotherapy in second line	Chemotherapy in second line	p
Total, n	100	25	75	
Median age(range), y	57.6 (21–80)	57.2 (30–74)	57.8 (21–80)	
Age group, n (%)				1
<65 years	68 (68.0)	17 (68.0)	51 (68.0)	
≥65 years	32 (32.0)	8 (32.0)	24 (32.0)	
Gender, n (%)				1
Male	40 (40.0)	10 (40.0)	30 (40.0)	
Female	60 (60.0)	15 (60.0)	45 (60.0)	
Smoking history, n (%)				0.32
Never-smoker	86 (86.0)	20 (80.0)	66 (88.0)	
Former/current smoker	14 (14.0)	5 (20.0)	9 (12.0)	
ECOG performance status, n (%)			0.73	
0	43 (43.0)	10 (40.0)	33 (44.0)	
1	57 (57.0)	15 (60.0)	42 (56.0)	
Pathological classification, n (%)				0.39
Adenocarcinoma	92 (92.0)	24 (96.0)	68 (90.7)	
NSCLC NOS	8 (8.0)	1 (4.0)	7 (9.3)	
TNM stage, n (%)				0.16
III	9 (9.0)	4 (16.0)	5 (6.7)	
IV	91 (91.0)	21 (84.0)	70 (93.3)	
EGFR mutation type, n (%)				1
19DEL	52 (52.0)	13 (52.0)	39 (52.0)	
L858R	48 (48.0)	12 (48.0)	36 (48.0)	
Type of EGFR-TKIs, n (%)				0.07
Gefitinib	44 (44.0)	16 (64.0)	28 (37.3)	
Erlotinib	19 (19.0)	3 (12.0)	16 (21.3)	
Icotinib	37 (37.0)	6 (24.0)	31 (41.3)	
PFS to EGFR-TKIs				1
<10 months	52 (52.0)	13 (52.0)	39 (52.0)	
≥10 months	48 (48.0)	12 (48.0)	36 (48.0)	
Distant metastasis, n(%)				0.13
Yes	82 (82.0)	23 (92.0)	59 (78.7)	
No	18 (18.0)	2 (8.0)	16 (21.3)	
Local therapy, n (%)				0.74
Yes	14 (14.0)	4 (16.0)	10 (13.3)	
No	86 (86.0)	21 (84.0)	65 (86.7)	

Same as in **Table 1**.

**Figure 5:**
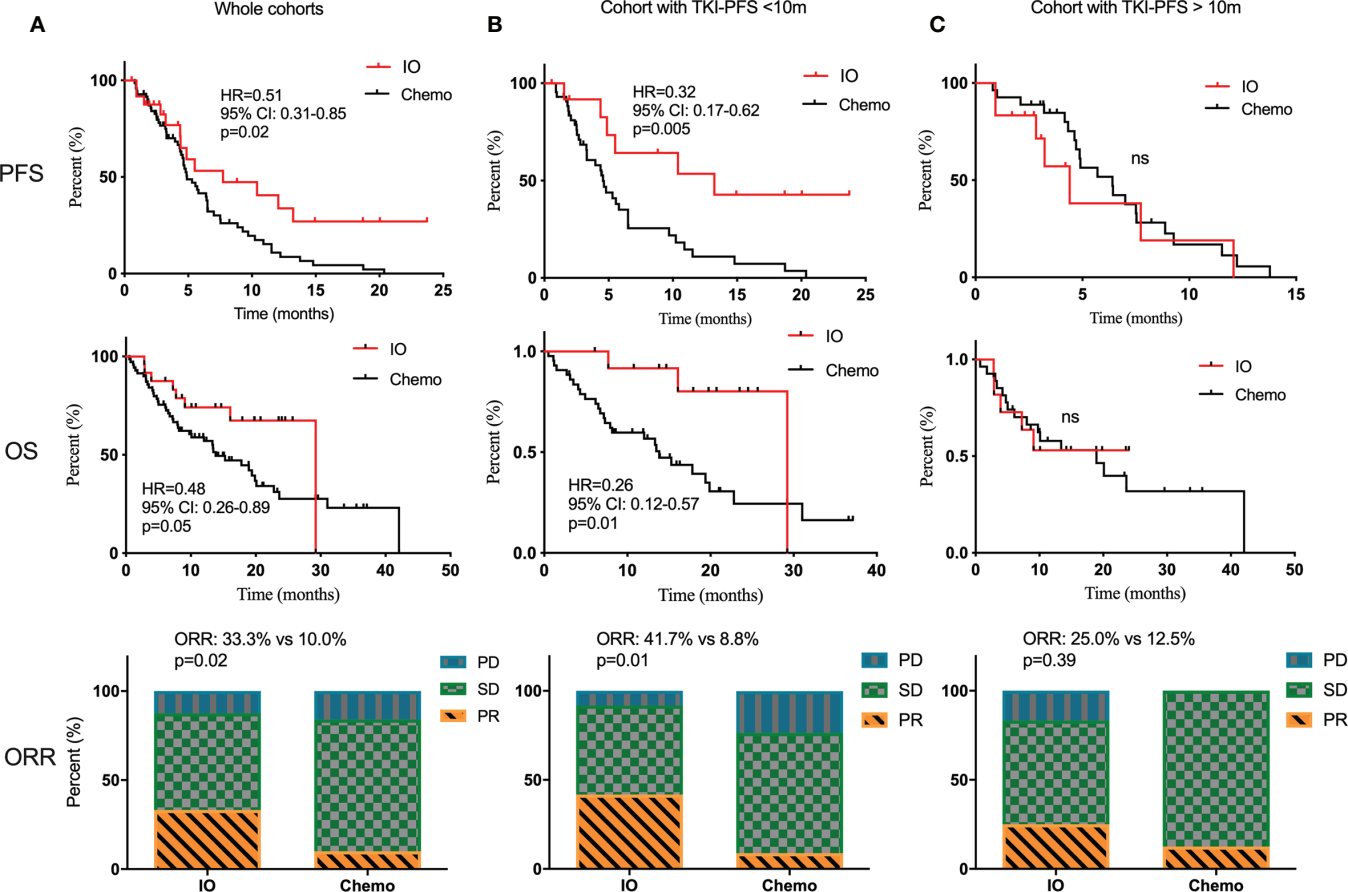
The comparison of traditional chemotherapy versus combined immunotherapy in second line treatment. The PFS, OS and ORR of traditional chemotherapy and combined immunotherapy in second line treatment among the whole cohorts **(A)**, cohort with TKI-PFS < 10 months **(B)**, and cohort with TKI-PFS > 10 months **(C)**. ns, none sense.

First of all, there was no difference in PFS and OS in the Chemo-cohort when grouping by TKI-PFS cutoff of 10 months (**Supplementary Figure 3**). Then we conducted a series of comparative analysis about PFS, OS, and ORR between the Immune-cohort and Chemo-cohort. Not surprisingly, combined immunotherapy demonstrated a significantly longer PFS (HR, 0.51, 95% CI: 0.31–0.85, p = 0.02), longer OS (HR, 0.48, 95% CI: 0.26–0.89, p = 0.05), and higher ORR (33.3 *vs* 10.0%, p = 0.02) than traditional chemotherapy (**Figure 5A**). Interestingly, stratified analysis by TKI-PFS found that the superiority of IO combined treatment only existed on the patients with short TKI-PFS (**Figure 5B**), with median PFS of 13.2 *vs* 4.5 months, HR, 0.32, 95% CI: 0.17–0.62, p = 0.005, median OS of 29.2 *vs* 13.8 months, HR, 0.26, 95% CI: 0.12–0.57, p = 0.01 and ORR, 41.7 *vs* 8.8%, p = 0.01, but not on the patients with long TKI-PFS (**Figure 5C**).

## Discussion

This study retrospectively investigated the association of targeted therapy with the outcomes of immunotherapy in advanced NSCLC patients with common EGFR sensitive mutation, and firstly identified a subgroup who could be most likely to benefit from IO combination treatment. As the results showed, patients with short TKI-PFS had statistically prolonged PFS and higher ORR from subsequent immunotherapy than those with longer TKI-PFS. And we firstly validated that the immune components and immune checkpoints in the TME were significantly different between those two cohorts, which provided an important new insight into the biological process of disease progression during EGFR-TKI target therapy. The strictly matched case–control study also suggested that anti-PD-1/PD-L1 based combination therapy possibly provided promising treatment models for this population.

Predictive biomarkers for ICI based immunotherapy had been explored for decades. Direct assessment of PD-L1 expression on tumor cells was a logical biomarker for the prediction of treatment effects on immunotherapy. Improved PFS and overall survival (OS) had been shown in patients with advanced NSCLC when comparing PD-L1 positive *versus* PD-L1 negative subgroups (7, 24, 27, 28). Previous studies found that patients with EGFR mutated type were less likely to be PD-L1-positive than EGFR wild type (29, 30), while other studies reported that EGFR mutation could stimulate the expression of PD-L1 (31, 32). Shan Su et al. reported that strong PD-L1 expression significantly decreased the ORR of EGFR-TKI treatment, and a high proportion of PD-L1 expression was found among patients with *de novo* resistance (17). It was not a unique instance, but had its counterpart. Another study involving 153 Taiwanese patients concluded that lower pre-treatment PD-L1 was associated with better ORR and PFS in EGFR mutated NSCLC treated with EGFR-TKIS, and patients with PD-L1 TPS ≥50% were more likely had primary resistance to EGFR-TKI (33). A phase 2 study evaluating the safety and efficiency of durvalumab as third-line treatment or later involved 111 of EGFR+/ALK+ NSCLC patients with PD-L1 expression layered at 25%, and it found that the clinical activity of durvalumab was encouraging in patients with ≥25% of tumor cells expressing PD-L1 (34). In our study, the prevalence of PD-L1 expression at disease progression was 50.0 and 17.6% by TPS ≥1% and ≥50%, which was slightly higher than the baseline data reported in the above studies, supporting the finding that six of 15 (40%) cases had increased PD-L1 expressions at disease progression (33). Patients with short TKI-PFS had a numerically higher rate of PD-L1 positive expression than those with long TKI-PFS, despite not reaching a statistical significance. Taking PD-L1 expression into consideration, there was no difference observed between PD-L1 positive and negative group whether among the whole cohort, cohort with short TKI-PFS, or cohort with long TKI-PFS, which might partly be biased by the limitation of small samples. Conversely, both in PD-L1 positive and negative cohorts, patients with short TKI-PFS had a statistically prolonged IO-PFS comparing with those who had long TKI-PFS. In addition, three patients with negative PD-L1 expression achieved IO-PFS longer than 10 months in the short TKI-PFS group while none in the long group. Hence, patients with short TKI-PFS were more likely to benefit from the subsequent IO combined treatment after EGFR-TKI treatment, which may be partially attributed to the higher PD-L1 expression level; but other mechanisms certainly exist.

Required T790M mutation was reported to have negative correlation with the efficiency of immunotherapy after EGFR-TKI treatment (25). Ching-Yao Yang and Byung Woo Yoon found that patients with longer TKI-PFS were more likely to acquire T790M resistance (33, 35). In our study, the frequency of required T790M mutation was 28.1% in the long TKI-PFS group, numerically higher than that in the short TKI-PFS group. However, subgroup analysis did not reach significant difference in response to ICI based therapy, which may partly be attributed to the relatively small proportion of T790M mutation in the whole cohort (13/58) as patients without T790M mutation were more likely to receive immunotherapy.

Preclinical studies reported that radiotherapy had synergistic effects with immunotherapy by increasing tumor antigen release, improving antigen presentation, and promoting lymphocytes infiltration (36–38). Clinical trials that evaluated the efficiency and safety of pembrolizumab after local radiotherapy for patients with advanced NSCLC concluded that pembrolizumab after radiotherapy could effectively improve PFS with good tolerance (39, 40). Narek Shaverdian’s et al. also found that previous radiotherapy in patients with advanced NSCLC resulted in longer PFS and OS with pembrolizumab treatment than those without (41). However, few studies had investigated it among NSCLC patients with EGFR-drive mutation. In our study, there were 11 patients (19%) who received local therapy before immunotherapy. No difference was found between patients with or without radiotherapy in the whole cohort, same as the result from univariate analysis. However, when taking TKI-PFS into consideration, we found that patients with short TKI-PFS were more likely to benefit from radiotherapy [**Supplementary Figure 4** **(i–v)**]. Larger trial is necessary to determine whether radiotherapy may assist immunotherapy in EGFR-mutated NSCLC, especially those with short TKI-PFS.

Tumor-host immune cells were considered to play crucial roles in tumor development and progression, and higher tumor infiltrated lymphocytes (TILs) were reported to be associated with improved survival in retrospective studies with a range of cancers such as colorectal cancer, melanoma, and NSCLC (42–45). Investigation of immune cell profiles provided further insights into the molecular underpinning of tumor progression. Previous studies demonstrated that patients with EGFR sensitive mutation showed significantly decreased T-cell infiltration than those with EGFR wild type, with the phenotype of low CD3/low KI67/low granzyme B and a shrinking proportion of PD-L1+/CD8+ TILs, which predicted poor response to immunotherapy (29, 42, 46). Our previous study explored the impact of EGFR-TKI on TME in EGFR-driven lung tumor models. It was found that after the use of sensitive EGFR-TKI, an increased cytotoxic CD8+ T cells and dendritic cells (DCs) dispelled Foxp3+ Tregs and inhibited M2-like polarization of macrophages which were observed in the early stage. However, this proinflammatory changes disappeared as treatment continued (47). In this study, we found that patients with short TKI-PFS had higher proportion of CD8+ effector T cells and proliferating T cells, and flow cytometry analysis validated those findings as short TKI-PFS cohort showed significantly higher proportion of CD3+ lymphocytes, CD8+ effector T cells and INF-*γ*+CD8+ cytotoxic T cells infiltrated in the TME. The concordance of increased proinflammatory cells strengthened the theoretical basis that patients with short TKI-PFS were more likely to benefit from combined immunotherapy. What’s more, the proportion of Foxp3+ in CD4+ T cells was significantly higher in patients with short TKI-PFS by flow cytometry, which may be the result of a feedback from increased effector T cell activation (48). Moreover, the rate of M2-like macrophages to M1-like macrophages was significantly higher in the long TKI-PFS group than in the short TKI-PFS group, which was discovered in single cell RNA-seq and validated by flow cytometry. Evidence suggested that M1-like macrophages had the function of pro-inflammatory, cytotoxic, and anti-tumorigenic while M2-like macrophages could suppress the immune response, promote tumor development, and inhibit inflammatory reaction (49, 50). The higher ratio of M2- to M1-like macrophages in longer TKI-PFS cohort possibly contributed to a more suppressive tumor microenvironment, impairing the clinical efficacy from ICI treatments.

Finally, we conducted a strictly matched case–control study to compare the clinical efficacy of chemotherapy combined with immunotherapy *versus* traditional chemotherapy in second-line setting for EGFR mutated NSCLC patients, who progressed on EGFR-TKIs and without the required T790M mutation. The results indicated that immune-combined chemotherapy would be a more suitable choice in second-line treatment for patients with short TKI-PFS. However, the size of immune-cohort was a little bit small; larger scale prospective clinical trial is needed to further explore and validate.

Several limitations should be taken into consideration. Firstly, this was a retrospective study and selection bias cannot be avoided. Secondly, the status of PD-L1 and TME in baseline was not evaluated, which might raise confusion whether the difference between those two populations exists initially or is induced by EGFR-TKI treatment. Fortunately, we have already conducted a prospective study to explore it. Thirdly, TMB was considered as a predictive biomarker in patients given ICI treatment. High TMB was associated with improved survival in patients receiving anti-PD-1 or anti-PD-L1 therapy across a wide variety of cancer types (51–53). Previous studies found that there was a negative correlation between TMB and clinical outcomes in metastatic EGFR-driven NSCLC patients, and patients with high TMB were more likely to achieve shorter TKI-PFS (16, 54). However, the data of TMB in this study was not available, which will be further explored in our following work. Finally, the mechanisms are still obscure, which will be the focus in the following work.

## Conclusions

In conclusion, the present study demonstrated that the PFS of EGFR-TKIs is an independent predictive factor for subsequent ICI based immunotherapy in EGFR mutated patients after progression on targeted therapy. EGFR mutated NSCLC patients with TKI-PFS less than 10 months conferred better response following combined immunotherapy. Patients with short TKI-PFS had higher intratumorally cytotoxic lymphocyte infiltration and lower rate of M2-like macrophages to M1-like macrophages. ICI combined with chemotherapy could be a promising treatment model in second-line treatment for patients with short TKI-PFS. The underlying mechanism needs to be further explored.

## Data Availability Statement

The datasets presented in this study can be found in SRA repository. The deposition link: https://www.ncbi.nlm.nih.gov/sra/PRJNA698465. The accession number: PRJNA698465.

## Ethics Statement

The studies involving human participants were reviewed and approved by the Ethics Committees of Shanghai Pulmonary Hospital Affiliated with Tongji University and were carried out in accordance with the World Medical Association’s Declaration of Helsinki. The patients/participants provided their written informed consent to participate in this study.

## Author Contributions

Conception and design: SL, FW, and CaZ. Administrative support: CaZ, ChZ, and XL. Provision of study materials or patients: WL, JY, FZ, AX, BC, and SR. Collection and assembly of data: SL, YJ, KJ, RH, and MQ. Data analysis and interpretation: SL, FW, and JF. Manuscript writing: SL and FW. All authors contributed to the article and approved the submitted version.

## Funding

This study was supported in part by grants from the National Nature Science Foundation of China (N0.81871865, 81871006, 81874036), National R&D projects (2016YFC0902300), Shanghai Science and Technology Medical Guidance Project (16411964400), the Science and Technology Commission of Shanghai Municipality (No. 19411950300), and the Shanghai Key Clinical Department Construction Project of Shanghai Municipal Health Commission-Respiratory Medicine.

## Conflict of Interest

JF was employed by Singleron Biotechnologies.

The remaining authors declare that the research was conducted in the absence of any commercial or financial relationships that could be construed as a potential conflict of interest.

## Supplementary Material

Supplementary Figure 1 The PFS of EGFR-TKI treatment **(A)** and immunotherapy **(B)** in the whole cohort.

Supplementary Figure 2 Subgroup analysis of IO-PFS according to the PD-L1 expression **(A)** and T790m status **(B)**.

Supplementary Figure 3 The PFS and OS of chemotherapy in second line treatment grouping by TKI-PFS.

Supplementary Figure 4 Subgroup analysis of IO-PFS according to local radiotherapy.
