# Supplementary figures and images for: Patients With Short PFS to EGFR-TKIs Predicted Better Response to Subsequent Anti-PD-1/PD-L1 Based Immunotherapy in EGFR Common Mutation NSCLC
Source: Front Oncol. 2021 Mar 11;11:639947. doi: 10.3389/fonc.2021.639947 (PMC7991800; doi:10.3389/fonc.2021.639947)

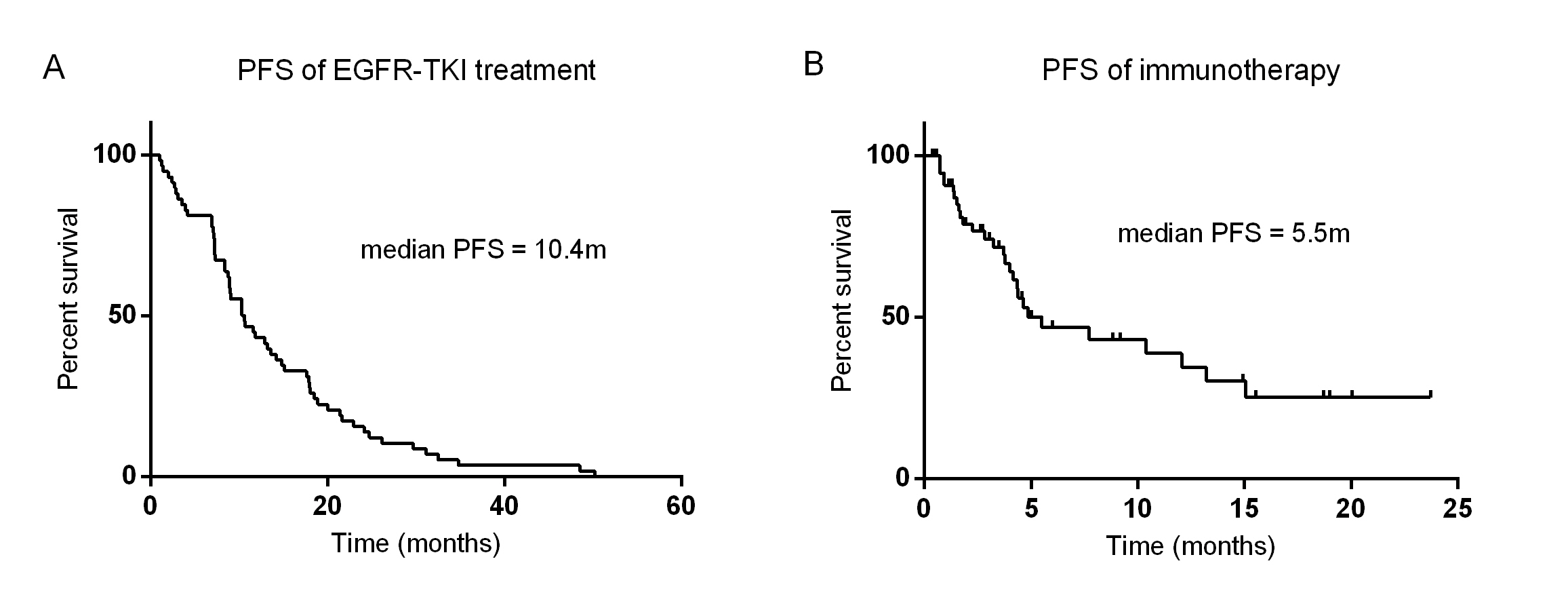

Supplement: Supplementary Figure 1 — The PFS of EGFR-TKI treatment (A) and immunotherapy (B) in the whole cohort. [file Image_1.jpeg]

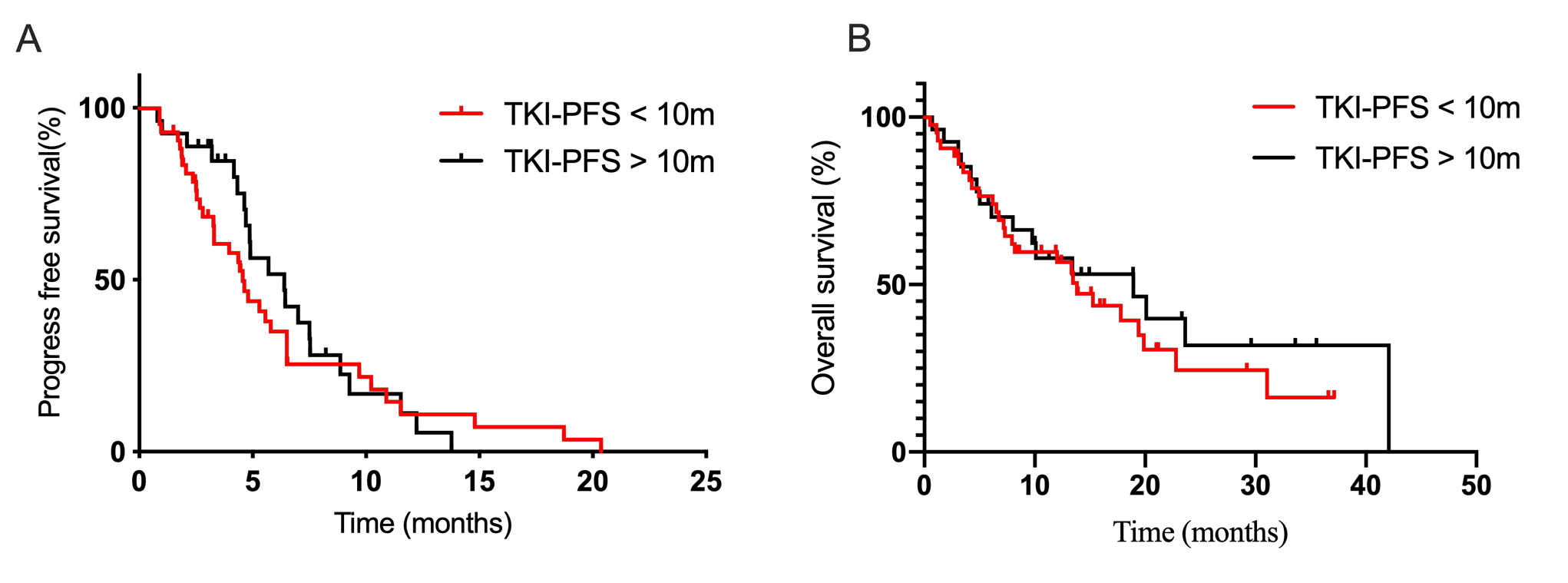

Supplement: Supplementary Figure 2 — Subgroup analysis of IO-PFS according to the PD-L1 expression (A) and T790m status (B). [file Image_2.tiff]

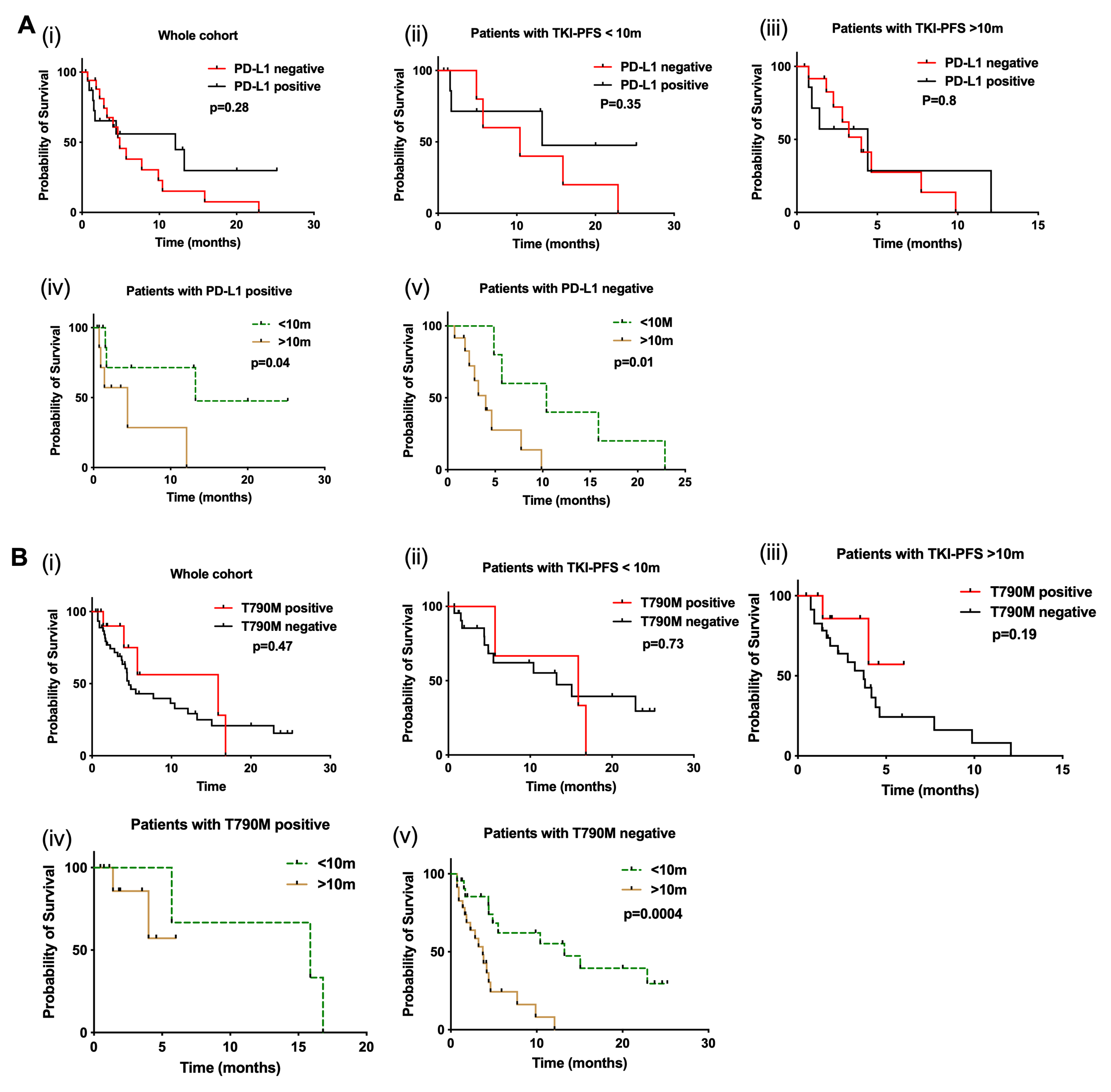

Supplement: Supplementary Figure 3 — The PFS and OS of chemotherapy in second line treatment grouping by TKI-PFS. [file Image_3.tiff]

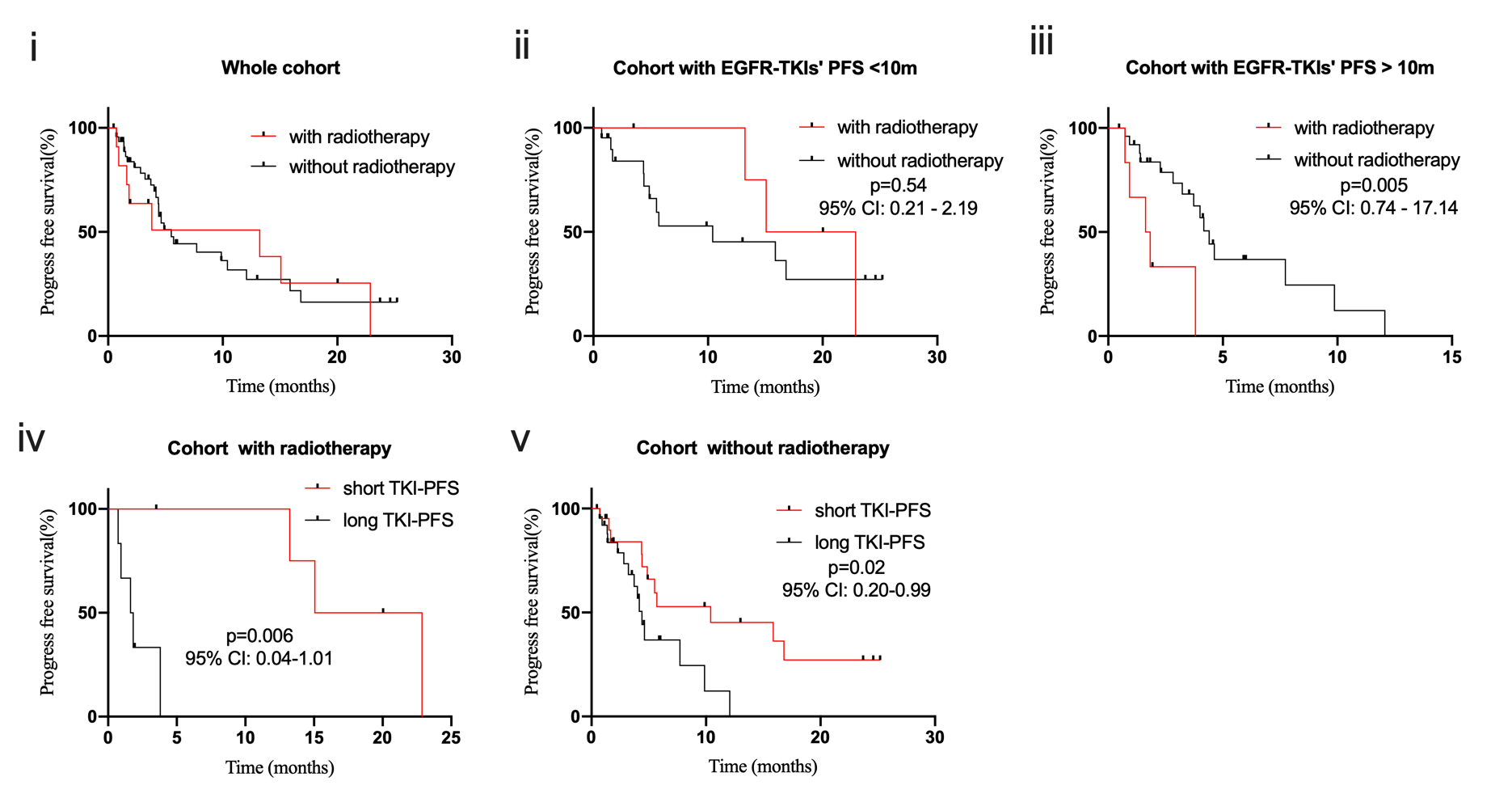

Supplement: Supplementary Figure 4 — Subgroup analysis of IO-PFS according to local radiotherapy. [file Image_4.tiff]
