# Supplementary material for: Patients With Short PFS to EGFR-TKIs Predicted Better Response to Subsequent Anti-PD-1/PD-L1 Based Immunotherapy in EGFR Common Mutation NSCLC
Source: Front Oncol. 2021 Mar 11;11:639947. doi: 10.3389/fonc.2021.639947 (PMC7991800; doi:10.3389/fonc.2021.639947)
Supplement: Supplementary file 5 [file Table_1.docx]

| Supplement Table 1. Basic information of patients for single cell RNA sequencing | | | | | | | | | | |
| --- | --- | --- | --- | --- | --- | --- | --- | --- | --- | --- |
| **Patients Number** | **Sex** | **Age** | **Smoking history** | **PS** | **EGFR type** | **Pathological type** | **Stage** | **Type of EGFR-TKI** | **TKI-PFS (d)** | **PD-L1 status** |
| No.1 | Male | 64 | Yes | 1 | 19DEL | Adeno | IV | Gefitinib | 104 | - |
| No.4 | Male | 51 | No | 0 | L858R | Adeno | IV | Erlotinib | 152 | 90% |
| No.6 | Female | 43 | No | 1 | L858R | Adeno | IV | Icotinib | 216 | 30+ |
| No.7 | Female | 57 | No | 1 | 19DEL | Adeno | IV | Erlotinib | 250 | Negative |
| No.8 | Female | 56 | No | 1 | 19DEL | Adeno | IV | Icotinib | 256 | Negative |
| No.9 | Female | 56 | No | 0 | L858R | Adeno | IV | Gefitinib | 329 | 10% |
| No.11 | Female | 64 | No | 1 | L858R | Adeno | IV | Gefitinib | 440 | - |
| No.13 | Male | 62 | No | 0 | 19DEL | Adeno | IV | Gefitinib | 516 | - |
| No.15 | Male | 68 | No | 1 | L858R | Adeno | IV | Erlotinib | 631 | - |
| No.16 | Male | 70 | No | 0 | 19DEL | Adeno | IV | Gefitinib | 667 | Negative |
| No.17 | Male | 65 | No | 1 | 19DEL | Adeno | IV | Erlotinib | 842 | 10-50% |
| No.18 | Female | 63 | No | 1 | 19DEL | Adeno | IV | Gefitinib | 1115 | Negative |

| Supplement Table 2. Basic information of patients for flow cytometry | | | | | | | | | | |
| --- | --- | --- | --- | --- | --- | --- | --- | --- | --- | --- |
| **Patients Number** | **Sex** | **Age** | **Smoking history** | **EGFR type** | **Pathological type** | **Stage** | **PS** | **TKI-PFS (d)** | **TKI recipe** | **PD-L1 status** |
| No.1 | Male | 69 | Yes | 19DEL | Adeno | IV | 1 | 34 | Icotinib | - |
| No.2 | Female | 69 | No | 19DEL | Adeno | IV | 0 | 76 | Gefitinib | 5% |
| No.3 | Male | 70 | No | L858R | Adeno | IV | 0 | 80 | Erlotinib | 90% |
| No.4 | Male | 72 | No | L858R | Adeno | IV | 0 | 93 | Gefitinib | Negative |
| No.5 | Female | 64 | No | L858R | Adeno | IIIB | 1 | 111 | Icotinib | 30+ |
| No.6 | Male | 43 | Yes | 19DEL | Adeno | IV | 0 | 143 | Gefitinib | Negative |
| No.7 | Female | 62 | No | 19DEL | Adeno | IV | 1 | 147 | Gefitinib | Negative |
| No.8 | Female | 66 | No | 19DEL | Adeno | IV | 1 | 195 | Icotinib | Negative |
| No.9 | Male | 55 | No | 19DEL | NSCLC | IV | 0 | 198 | Gefitinib | - |
| No.10 | Male | 55 | Yes | L858R | Adeno | IV | 1 | 230 | Gefitinib | 1-5% |
| No.11 | Female | 61 | No | 19DEL | Adeno | IIIB | 0 | 232 | Gefitinib | 60% |
| No.12 | Female | 61 | No | 19DEL | Adeno | IV | 0 | 260 | Icotinib | - |
| No.13 | Female | 73 | No | L858R | Adeno | IV | 1 | 263 | Gefitinib | Negative |
| No.14 | Male | 69 | Yes | 19DEL | Adeno | IV | 0 | 323 | Gefitinib | - |
| No.15 | Female | 55 | No | L858R | Adeno | IV | 0 | 343 | Gefitinib | 10% |
| No.16 | Female | 46 | No | L858R | Adeno | IV | 1 | 370 | Gefitinib | - |
| No.17 | Female | 56 | No | 19DEL | Adeno | IV | 0 | 380 | Gefitinib | - |
| No.18 | Male | 69 | No | 19DEL | Adeno | IV | 0 | 398 | Gefitinib | - |
| No.19 | Male | 51 | No | L858R | Adeno | IV | 1 | 408 | Erlotinib | - |
| No.20 | Female | 44 | Yes | L858R | Adeno | IV | 0 | 441 | Icotinib | Negative |
| No.21 | Male | 62 | No | 19DEL | Adeno | IV | 0 | 467 | Gefitinib | Negative |
| No.22 | Male | 67 | No | 19DEL | Adeno | IV | 1 | 478 | Erlotinib | 10-50% |
| No.23 | Female | 64 | No | 19DEL | Adeno | IV | 1 | 578 | Gefitinib | Negative |
| No.24 | Male | 37 | No | 19DEL | NSCLC | IIIB | 0 | 635 | Gefitinib | Negative |
| No.25 | Male | 62 | No | L858R | Adeno | IV | 0 | 712 | Icotinib | - |
| No.26 | Female | 62 | No | 19DEL | Adeno | IV | 0 | 805 | Gefitinib | Negative |
